# Prefrontal θ-Burst Stimulation Disrupts the Organizing Influence of Active Short-Term Retrieval on Episodic Memory

**DOI:** 10.1523/ENEURO.0347-17.2018

**Published:** 2018-02-13

**Authors:** Bianca M. Marin, Stephen A. VanHaerents, Joel L. Voss, Donna J. Bridge

**Affiliations:** Department of Medical Social Sciences, Ken and Ruth Davee Department of Neurology, Department of Psychiatry and Behavioral Sciences, and Interdepartmental Neuroscience Program, Northwestern University Feinberg School of Medicine, Chicago, IL 60611

**Keywords:** active retrieval, dorsolateral prefrontal cortex, long-term memory, spatial memory, θ-burst, TMS

## Abstract

Dorsolateral prefrontal cortex (DLPFC) is thought to organize items in working memory and this organizational role may also influence long-term memory. To causally test this hypothesized role of DLPFC in long-term memory formation, we used θ-burst noninvasive stimulation (TBS) to modulate DLPFC involvement in a memory task that assessed the influence of active short-term retrieval on later memory. Human subjects viewed three objects on a grid and then either actively retrieved or passively restudied one object’s location after a brief delay. Long-term memory for the other objects was assessed after a delay to evaluate the beneficial role of active short-term retrieval on subsequent memory for the entire set of object locations. We found that DLPFC TBS had no significant effects on short-term memory. In contrast, DLPFC TBS impaired long-term memory selectively in the active-retrieval condition but not in the passive-restudy condition. These findings are consistent with the hypothesized contribution of DLPFC to the organizational processes operative during active short-term retrieval that influence long-term memory, although other regions that were not stimulated could provide similar contributions. Notably, active-retrieval and passive-restudy conditions were intermixed, and therefore nonspecific influences of stimulation were well controlled. These results suggest that DLPFC is causally involved in organizing event information during active retrieval to support coherent long-term memory formation.

## Significance Statement

Although dorsolateral prefrontal cortex (DLPFC) has been implicated in short-term working memory organization, its long-term memory contributions have not been established. Building on fMRI findings, we tested the role of DLPFC in organizing event information during memory formation to support long-term episodic memory. Our task involved both active-retrieval and passive-restudy encoding conditions, which varied the extent to which event elements were organized around select information. Modulation of DLPFC via θ-burst noninvasive stimulation (TBS) selectively altered long-term memory formation in the active-retrieval condition, but not the passive-restudy condition. These results pinpoint the role of DLPFC in organizing event information to form coherent long-term memories and demonstrate that TBS can be used to disentangle cognitive processes that contribute to long-term memory.

## Introduction

The hippocampus is essential for binding the various elements that comprise events to form long-term memory representations (Bunsey and Eichenbaum, 1996; Eichenbaum and Cohen, 2001; Konkel et al., 2008; Giovanello et al., 2009). However, much is unknown regarding how event information is structured into coherently organized memory representations (Bridge and Voss, 2015; Bridge et al., 2017). The prefrontal cortex could contribute to memory organization, as it interacts with the hippocampus during long-term memory encoding and retrieval (Blumenfeld and Ranganath, 2006; Murray and Ranganath, 2007; Hannula and Ranganath, 2009; Blumenfeld et al., 2011; Voss et al., 2011). The lateral and frontopolar aspects of prefrontal cortex have been implicated in the organization of information in working memory and in the allocation of attention to goal-relevant event features (D'Esposito et al., 1999; Postle et al., 1999, 2006; D'Esposito and Postle, 2015). Prefrontal selection processes could therefore serve to structure event information so that it is bound by the hippocampus in an organized way, enabling the formation of coherent memory representations (Blumenfeld and Ranganath, 2006; Blumenfeld et al., 2011).

Previous studies have shown that active short-term memory retrieval of one element from a multi-element event causes subsequent long-term memories to become organized around the retrieved element (Bridge and Voss, 2015; Bridge et al., 2017). In those studies, retrieved elements later served as superior retrieval cues for other event elements, indicating the memory was coherently structured around the retrieved elements. Furthermore, this beneficial effect of short-term retrieval on coherent memory formation was associated with interactivity between the hippocampus and a frontoparietal set of regions that included the dorsolateral prefrontal cortex (DLPFC) as measured via fMRI (Bridge et al., 2017). In the current study, we used θ-burst transcranial magnetic stimulation (θ-burst noninvasive stimulation; TBS) to test the role of DLPFC in memory organization, using a modification of the memory paradigm that previously identified prefrontal-hippocampal interactions relevant for long-term memory organization (Bridge et al., 2017).

Other studies have used TBS to test the role of prefrontal cortex in long-term memory (e.g., Lee et al., 2013; Blumenfeld et al., 2014; Ryals et al., 2015). However, those studies have focused on general memory constructs (i.e., overall accuracy and response confidence) without specifically testing the role of DLPFC in long-term memory organization. In previous studies on working memory, transcranial magnetic stimulation (TMS) of the prefrontal cortex has modulated its interaction with posterior cortical regions responsible for perceptual representation, thereby modulating working-memory accuracy (Zanto et al., 2011; Lorenc et al., 2015). Likewise for long-term memory, prefrontal stimulation has been shown to alter interactions with the hippocampus in relation to task performance (Bilek et al., 2013). Although fMRI studies have been used to identify the intersection between organizational processes during working-memory and subsequent long-term memory representations in the DLPFC and hippocampus (Blumenfeld and Ranganath, 2006; Murray and Ranganath, 2007; Blumenfeld et al., 2011; Bridge et al., 2017), no study has tested the causal role of DLPFC in this process using noninvasive stimulation.

The current study followed previous research on the role of DLPFC organizational and selection processes that contribute to long-term memory (Bridge and Voss, 2015; Bridge et al., 2017), but with modifications to accommodate TBS. In those previous experiments, subjects studied many events each comprised of three objects at specific locations. Following a brief delay after each event, subjects either selected an object and retrieved its location (active retrieval) or moved an object to its location indicated by a visual cue (passive restudy). During delayed memory testing, subjects were given one object as a reminder cue and memory for one of the non-manipulated objects was tested. The major behavioral finding in those studies was that subjects recalled the non-manipulated object locations more accurately when the actively retrieved objects served as the reminder cue, compared to when the passively moved objects served as the reminder cue. Thus, objects that subjects selected for active short-term retrieval became strongly bound to the rest of the objects within an event, and therefore served as strong reminder cues for the entire event episode. Furthermore, fMRI findings indicated that the active manipulation condition involved interactions between the DLPFC and the hippocampus that were not seen in the passive manipulation condition and that were related to later long-term memory (Bridge et al., 2017). These results indicate that active retrieval as measured by this experiment design has an organizational influence on memory content, with the long-term memory content coherently organized around the actively retrieved content. Further, this process is associated with DLPFC-hippocampus interactions during active retrieval.

The current experiment follows the general logic of these previous experiments (Bridge and Voss, 2015; Bridge et al., 2017), but with TBS used to influence DLPFC function. A between-group design was used, whereby each subject completed versions of the aforementioned memory task on two separate days. One group received sham (near-zero intensity) TBS before task performance on both days (sham group) and the other group received sham TBS before task performance on the first day then active (full-intensity) TBS before task performance on the second day (stim group). This allowed us to compare each subject’s performance following active or sham TBS to their performance at the sham-intensity baseline session. Based on the considerations reviewed above, particularly the previous evidence that DLPFC-hippocampal interactions are relevant for the effects of active retrieval on subsequent long-term memory (Bridge et al., 2017), we hypothesized that TBS of the DLPFC would disproportionately affect memory for objects in the active-retrieval condition. The experiment design permitted assessment of TBS effects on both short-term memory and long-term memory, such that the role of DLPFC in the long-term memory organization process could be more precisely specified.

## Materials and Methods

### Subjects

Data were collected from 30 healthy adults (18 women; ages 18–38 years). Subjects were randomly assigned in equal numbers to the sham group and the stim group. Two subjects, one from each group, were excluded from all analysis. One subject was excluded due to poor performance on the short-term spatial memory task at the first session (performance fell >2 SDs below the mean: M = 0.27) and the second subject was excluded due to poor performance on the associative recognition test at the first session (M = 0.41), leaving *n* = 14 per group for the final analyses (sham: seven female, mean age = 25.1 years; stim: nine female, mean age = 28.1 years). No subjects reported neurologic or psychiatric disorders or the current use of psychotropic drugs. All subjects were deemed eligible for MRI and TMS procedures based on standard safety screenings (Rossi et al., 2009) overseen by a board-certified neurologist (S.A.V.). All subjects gave written consent and were remunerated for their participation. The study was approved by the Institutional Review Board.

### Design overview

Subjects completed two memory testing sessions with an ∼48-hour interval between sessions (41–55 h). Subjects were randomly assigned to either the stim or sham condition. session 1 was identical across subject groups and served as a baseline for session 2 performance. At session 1, all subjects received sham-intensity TBS, which was set to 20% of the subject’s motor threshold (see below). TBS took ∼40 s. Immediately following stimulation (within 5 min), subjects began the memory task, including three study-test blocks. Testing was completed within 60 min of stimulation (within the generally accepted after-effect period for TBS; Thut & Pascual-Leone, 2010). Session 2 was identical to session 1, but with different stimulation intensity for the stim group. Subjects in the sham condition received the same stimulation protocol as during session 1, whereas subjects in the stim group received TBS at 80% motor threshold. Following stimulation, subjects completed the same memory task as on the first session but with a different set of stimuli.

### MRI

**Figure 1.**
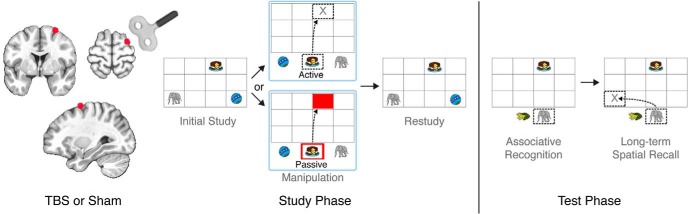
Experimental design overview. At each experimental session, subjects received full-intensity TBS or sham-intensity TBS to an area of right DLPFC identified via an fMRI experiment using similar memory testing conditions (Bridge et al., 2017), as marked in red. Immediately following stimulation, subjects completed three study-test blocks. Active-retrieval and passive-restudy trials were randomly intermixed. For active-retrieval trials, subjects studied three objects (initial study; 4 s), then selected one object (∼1.8 s), and moved it to its associated location (∼1.1 s; manipulation). These trials were used to assess short-term spatial recall accuracy. For passive-restudy trials, subjects clicked on the highlighted object (∼1.3 s) and then dragged the experiment-determined object to its associated location (∼1.2 s), indicated by a red box on the grid. Subjects then continued to view all objects at their associated locations (restudy; 4 s). Following a 60-s distractor, subjects completed delayed memory testing. Subjects attempted to select the object at the bottom of the screen associated with the reminder cue on the grid, given the presence of a foil from a different trial (∼2.2 s; associative recognition) and then moved the object to its associated location (∼1.6 s; long-term spatial recall).

Structural MRI scans were acquired before the first session to provide subject-specific anatomic guidance for TMS. Structural MRI scans were obtained using a Siemens 3T TIM Trio whole-body scanner with a 32-channel head coil (MPRAGE T1-weighted scans, TR = 2400 ms, TE 3.16 ms, voxel size = 1 mm^3^, FOV = 25.6 cm, flip angle 8°, 176 sagittal slices). MRI data were processed using AFNI (Cox, 1996). Structural MRI data were transformed into stereotactic space using the Montreal Neurologic Institute (MNI-305) template (Evans et al., 1993), and the transformation matrix was stored to allow conversion between original and standardized spaces. The stimulation target was marked at MNI coordinate (+28, –1, +68) based on fMRI results from Bridge et al. (2017). This stimulation target was the posterior aspect of the superior frontal gyrus. This area showed increased activity at the group level immediately following active retrieval relative to passive re-exposure while subjects restudied the objects in their locations, and was therefore associated with active retrieval within the same experimental design as used here (Fig. 1). The normalized structural image with the target marked was then transformed back into original MRI space for localization during TBS. TBS was thus delivered to the same MNI coordinate in all subjects despite individual differences in neuroanatomy.

### TMS

TMS was delivered under stereotactic guidance using a Nexstim eXimia NBS 4.3 with a 70-mm figure-eight coil (Nexstim Ltd.). Passive motor threshold was determined as the minimum intensity required for single TMS pulses of the M1 thumb area to produce contraction of the abductor pollicis brevis of at least 50 mV for 6/10 consecutive trials, as measured by surface EMG (Rossini et al., 1994).

TBS involved three biphasic pulses at 50 Hz delivered every 200 ms (5 Hz) for 40 s, yielding 600 total biphasic pulses. Sham-intensity TBS was delivered at 20% of motor threshold (M = 7.1% maximum stimulator output). Active TBS for the Stim condition was delivered at 80% of motor threshold (M = 30.6% maximum stimulator output). For ten subjects (two stim, eight sham) who had motor thresholds for which 80% would have exceeded the maximum intensity of the Nexstim system for the θ-burst protocol, the maximum stimulation intensity for the system was applied for stim (M = 38% maximum stimulator output, equivalent to M = 64.8% of motor threshold for these subjects). For sham, 20% of the maximum stimulation intensity was used (M = 7.6% maximum stimulator output, equivalent to M = 16.84% of motor threshold for these subjects). Subjects received a test pulse before TBS, and were given the option to discontinue stimulation at any point if they found it aversive.

Although a potential concern with the sham TBS control condition is that lower intensity cannot produce the same sensations experienced with full-intensity TBS (Jung et al., 2016), it is important to note that hypotheses concerned effects of TBS on active-retrieval versus passive-restudy trials, which were intermixed. It is unlikely that any nonspecific physical sensations caused by full-intensity TBS would selectively influence performance for these trials. Testing was automated and performed after TBS administration was complete, and subjects were unaware of condition-specific hypotheses.

### Memory task

Two sets of 240 object images were used, for a total of 480 unique object images. One set comprised images of real-life objects (Brodeur et al., 2010), and the other set comprised color drawings of objects (Rossion and Pourtois, 2004). Each block contained unique objects, all from the same object set. Each object was encapsulated by a white box with dimensions 3.71 × 3.71 cm. Most of the screen was occupied by a green rectangular grid composed of 18 individual spaces separated by thick, black lines. Additional gray space at the bottom of the screen was reserved for the object recognition and spatial recall tests. Each object could appear in the center of any of the 18 grid spaces. The grid dimensions were 34.65 × 13.61 cm. The screen resolution was 1280 × 720 pixels on an LCD monitor, with a refresh rate of 60 Hz.

Subjects completed three study-test blocks. Each study block consisted of 10 active trials, 10 passive trials, and four catch trials, administered in intermixed, randomized order. During each active and passive trial, subjects studied three objects in unique locations on a grid (Fig. 1). After a brief distractor, one object was subjected to an active or passive manipulation (i.e., active retrieval vs passive restudy), in which all three objects appeared at the bottom of the grid, and subjects selected one object and move it to its associated location. The active and passive study trials were randomly intermixed during the Study phase of each block. Immediately following the manipulation, subjects restudied the objects in their associated locations. Following a block of 24 study trials, subjects completed the long-term memory test. One object from each study trial trio served as a reminder cue. Subjects selected the object associated with the reminder cue (out of two options) and then recalled its associated location.

Subjects initially studied the three object locations for 4000 ms. This was followed by a brief distractor task, during which subjects viewed two kaleidoscope images consecutively and had to decide whether the images were identical or different. After the distractor task, one object was subjected to an active or passive manipulation. In the active condition, subjects chose one object and recalled its associated grid location (active retrieval). This served as the short-term spatial memory test. If the object was placed in an incorrect location, it immediately moved to its correct location. In the passive condition, subjects moved the preselected object to its location on the grid indicated by a visual cue (passive restudy). The preselected object was highlighted by a thick red outline, and its associated location on the grid was indicated by a red box. Following the manipulation, subjects restudied all three objects in their correct locations for 4000 ms. A 1000-ms intertrial interval occurred between each study trial.

In addition to the active and passive trials, four randomly distributed catch trials occurred during the study phase. The catch trials served to encourage subjects to view all objects during the initial study period. On catch trials, subjects were required to recall one randomly selected object’s location after the initial study period. Subjects then received immediate feedback on their recall accuracy. Catch trials were not subsequently tested or analyzed.

Following each study block, subjects viewed pictures of cats during a 60-s delay. Subjects then completed the test phase. The test phase included 20 trials (catch trials were not included in the test phase). For each object trio from the study phase, one object (either manipulated or non-manipulated) appeared in its location on the grid, serving as a reminder cue for one of the associated objects from the same set. Manipulated objects were reminder cues on half of the trials and non-manipulated objects were reminder cues on the other half of the trials. Subjects were given two familiar object choices below the grid and were prompted to select the object that had been studied with the cue (associative recognition test), and then recall its associated location on the grid (long-term spatial memory test). Each object choice appeared on two separate test trials: once as the correct object choice associated with the cue, and once as a lure. Importantly, the tested objects were always non-manipulated objects.

## Results

### Short-term spatial memory

**Figure 2.**
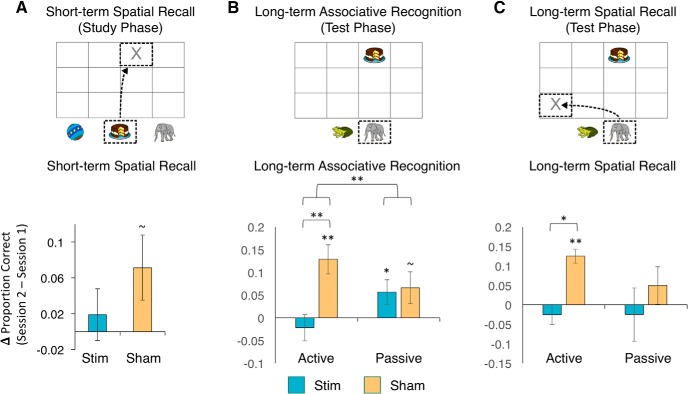
Short-term and long-term episodic memory following DLPFC stimulation. ***A***, Short-term spatial memory was not significantly altered due to DLPFC stimulation. ***B***, Long-term associative recognition memory was selectively reduced in the active-retrieval condition following DLPFC stimulation relative to sham. ***C***, Long-term spatial recall in the active-retrieval condition was also selectively impaired following DLPFC stimulation relative to sham. Error bars depict SEM. ∼*p* < 0.1, *p < .05, **p < .01.

During the Active retrieval study manipulation, subjects selected one object and recalled its associated location shortly after initially studying the objects. We measured short-term spatial memory at each session as the proportion of trials in which subjects placed the object its correct location during the Active retrieval study manipulation (Fig. 2*A*). The groups did not differ in session 1 accuracy [sham: M = 0.84, SE = 0.04; stim: M = 0.80, SE = 0.03; *t*_(26)_ = 0.53, *p* = 0.60]. To examine effects of stimulation on short-term spatial recall, we subtracted the session 1 baseline accuracy from session 2 accuracy for each group and compared this change in performance between groups. We conducted a one-way *t* test for each group to determine whether short-term recall performance changed significantly across testing sessions. Performance in the sham group tended to improve across sessions [*t*_(13)_ = 1.97, *p* = 0.07], whereas performance did not change across sessions for the stim group [*t*_(13)_ = 0.66, *p* = 0.52]. A two-way *t* test comparing the change in performance across groups indicated no differences across groups [*t*_(26)_ = 1.13, *p* = 0.27]. These results suggest that DLPFC stimulation produced no significant changes in the accuracy of short-term memory retrieval, despite a numerical trend for reducing a weak practice effect across sessions that was evident in the sham group in the absence of stimulation.

### Long-term associative recognition memory

We measured associative recognition accuracy by calculating the proportion of trials in which subjects selected the correct object associated with the reminder cue during the delayed memory test (Fig. 2*B*). For this analysis, we only considered trials in which the manipulated object was correctly placed in its original location during the study phase. Active [sham: M = 0.78, SE = 0.03; stim: M = 0.83, SE = 0.04; *t*_(26)_ = 0.89, *p* = 0.38] and passive [sham: M = 0.80, SE = 0.05; stim: M = 0.77, SE = 0.05; *t*_(26)_ = 0.49, *p* = 0.63] scores did not differ among groups at baseline during session 1. We computed the change in associative recognition accuracy due to stimulation by subtracting session 1 performance from session 2 performance for each group and for each condition. We conducted a two-way mixed ANOVA with stimulation condition (stim vs sham) as the between-subjects factor and study condition (active vs passive) as the within-subjects factor. This revealed a significant effect of stimulation [*F*_(1,26)_ = 5.15, *p* = 0.03]. However, this main effect was qualified by an interaction of stimulation and study conditions [*F*_(1,26)_ = 7.37, *p* = 0.01]. In the active condition, the change in associative recognition accuracy was significantly less following stim compared to sham stimulation [*t*_(26)_ = 3.47, *p* = 0.002]. However, changes in associative recognition performance in the passive condition did not vary for the stim and sham conditions [*t*_(26)_ = 0.21, *p* = 0.84]. These results indicate that DLPFC TBS selectively impaired long-term associative recognition for those trials that involved active retrieval during encoding.

We conducted follow-up one-way *t* tests to determine whether performance significantly changed across sessions for each group and study condition. For the active condition, associative recognition accuracy significantly improved across sessions for the sham group [*t*_(13)_ = 3.99, *p* = 0.002], whereas DLPFC stimulation blocked this memory improvement in the stim group [*t*_(13)_ = 0.75, *p* = 0.46]. These results were in stark contrast to the passive condition, in which both the sham group [*t*_(13)_ = 1.89, *p* = 0.08] and the stim group tended to improve across sessions [*t*_(13)_ = 2.11, *p* = 0.05]. These results show that DLPFC stimulation selectively impaired long-term memory performance for sets of objects that were encoded with the active retrieval manipulation. These results provide support for the hypothesis that (1) active retrieval promotes organization of event information to support coherent long-term memories and (2) DLPFC contributes to the active retrieval organization process.

### Long-term spatial recall

Immediately after subjects selected the associated object during the delayed memory test, they were prompted to place the selected object in its original location (Fig. 2*C*). We quantified long-term spatial memory accuracy as the proportion of trials in which subjects placed the associated object in its correct location.

We conducted a two-way mixed ANOVA with stimulation condition (stim vs sham) as the between-subjects factor and study condition (active vs passive) as the within-subjects factor. The main effect of stimulation condition was significant [*F*_(1,26)_ = 4.77, *p* = 0.04]; however, the interaction of Stimulation condition and study condition was not significant [*F*_(1,26)_ = 1.34, *p* = 0.26]. Importantly, baseline long-term spatial recall scores at session 1 did not differ across stimulation groups for the active condition [sham: M = 0.52, SE = 0.05; stim: M = 0.51, SE = 0.06; *t*_(26)_ = 0.05, *p* = 0.96] nor for the passive condition [sham: M = 0.56, SE = 0.04; stim: M = 0.55, SE = 0.06; *t*_(26)_ = 0.21, *p* = 0.83].

Given our hypothesis that DLPFC stimulation should selectively alter performance in the active condition, we conducted a priori *t* tests to determine whether the same pattern we observed for associative recognition accuracy was present for long-term spatial recall. Consistent with our hypothesis, long-term spatial recall accuracy was significantly worse following stim compared to Sham stimulation [*t*_(26)_ = 2.38, *p* = 0.02]. However, DLPFC stimulation did not significantly alter long-term spatial recall performance in the passive condition [*t*_(26)_ = 1.27, *p* = 0.21].

We conducted follow-up one-way *t* tests to determine whether long-term spatial memory performance significantly changed across sessions for each group and study condition. For the active condition, long-term spatial recall significantly improved across sessions for the sham group [*t*_(13)_ = 2.95, *p* = 0.01], whereas performance did not change across sessions for the stim group [*t*_(13)_ = 0.54, *p* = 0.60]. On the other hand, for the passive condition, neither the sham group [*t*_(13)_ = 1.09, *p* = 0.30] nor the stim group [*t*_(13)_ = 0.68, *p* = 0.51] showed improvements in long-term spatial recall across sessions. These results suggest that the active retrieval manipulation during study enabled subjects to improve long-term spatial memory performance across sessions, whereas the passive condition did not enable any such improvement, perhaps because subjects were able to implement a more effective retrieval-induced organizational strategy after practicing the task at session 1 in the sham group. Furthermore, these results provide more support for the hypothesis that DLPFC is selectively and causally involved in effectively organizing event information to support the formation of coherent long-term memory representations.

## Discussion

Previous studies have shown that active retrieval of select information during learning promotes the formation of coherent memory representations by causing event information to become organized around the retrieved content. Furthermore, an fMRI study showed that DLPFC is involved in this retrieval-induced organizational process (Bridge et al., 2017). Here, we tested whether DLPFC plays a critical role in organizing relational elements to support long-term memory formation. To do this, we applied θ-burst stimulation to the DLPFC of subjects before completing a memory task that involved both active-retrieval and passive-restudy learning conditions. Despite no significant effects on short-term retrieval accuracy, TBS had robust effects on long-term associative recognition memory and spatial recall in the active-retrieval condition, but not in the passive-restudy condition. Importantly, we focused only on those trials for which short-term recall was accurate, and so effects on long-term memory were not likely secondary to effects on short-term memory (coupled with the fact that effects on short-term memory were not significant). Thus, DLPFC stimulation did not merely disrupt long-term relational memory, but it disrupted the beneficial effects of short-term active retrieval on long-term memory formation. We hypothesize that DLPFC stimulation impaired the organizational processes that are typically engaged following active retrieval. As a consequence, memory of the entire episode was disrupted in the active condition. On the other hand, because these organizational processes are not actively engaged following passive restudy, memory performance remained intact following stimulation for this condition. Our within-subjects manipulation of study condition (active/passive) thus yielded high specificity regarding the influence of stimulation on DLPFC contributions to long-term memory.

Although substantial research has shown that DLPFC is causally involved in working memory organization, no studies have shown that it is necessary for organizational processes that promote coherent long-term memory formation. It is interesting that the effects of stimulation were minimal on short-term spatial recall, although DLPFC significantly modulated long-term memory for events encoded within this active retrieval study condition. We hypothesize that the DLPFC contribution to long-term memory organization occurred immediately following active retrieval, during the ensuing restudy period. Indeed, these results are consistent with fMRI results showing enhanced DLPFC activity following active retrieval, but not during retrieval (Bridge et al., 2017). During the restudy interval, we have shown that active retrieval influences the pattern of exploratory eye movements subjects engage in, and that these strategic eye movement patterns contribute to long-term memory. Specifically, following active retrieval, subjects divert their attention away from the retrieved element and focus attention on the other non-retrieved elements. This pattern of exploratory eye movements promoted long-term memory and involved coordination between hippocampus and DLPFC. Future studies should use eye-movement tracking paired with TMS to determine whether DLPFC is causally linked to exploration during learning.

There have been some inconsistent findings regarding the influence of TBS on memory function. Whereas some studies have shown that TBS applied to the prefrontal cortex disrupts long-term memory (Blumenfeld et al., 2014), others have shown TBS prefrontal stimulation enhances long-term memory in some conditions (Lee et al., 2013; Blumenfeld et al., 2014; Ryals et al., 2015). These studies stimulated different prefrontal locations and measured effects using different task formats, which may have contributed to divergent results. Indeed, TBS stimulation effects vary considerably across studies using different stimulation locations and cognitive measures (Demeter, 2016). Likewise, TBS effects on prefrontal cortex are likely complex and poorly understood. It is unlikely that TBS influenced only the stimulation location of DLPFC, given that noninvasive stimulation is generally thought to influence activity within distributed networks (Fox et al., 2012), and prefrontal TBS has been shown to influence widespread prefrontal connectivity (Gratton et al., 2013). It is possible that the functional network including DLPFC and hippocampus was influenced by stimulation, as studies have shown that this network is critical for the effects of the active-retrieval condition on memory organization using a variant of the current paradigm (Bridge et al., 2017), and previous studies have shown that prefrontal TMS can change hippocampal activity during memory-related processing (Bilek et al., 2013). Importantly, our results do not show selective involvement of DLPFC in organizing memories during active learning, as we did not stimulate any other regions. Indeed, based on previous fMRI findings (Bridge et al., 2017), portions of the parietal cortex may work together with DLPFC to support memory organization. However, we do show that DLPFC is involved specifically in the active retrieval learning condition and not the passive restudy learning condition, demonstrating that it is crucially involved in organizational processes during learning that support long-term memory.

In conclusion, the findings suggest that DLPFC TBS causally influences the organizational processes occurring during active retrieval that structure long-term memory. These results support the previously established role of DLPFC in organizing event information. The study shows that TBS can be successfully employed to investigate the DLPFC contributions to complex memory functions, and lays a solid foundation for future studies looking at the role of DLPFC in creating coherent episodic memories.

## Synthesis

Reviewing Editor: Bradley Postle, University of Wisconsin Decisions are customarily a result of the Reviewing Editor and the peer reviewers coming together and discussing their recommendations until a consensus is reached. When revisions are invited, a fact-based synthesis statement explaining their decision and outlining what is needed to prepare a revision will be listed below. The following reviewer(s) agreed to reveal their identity: Paula Croxson.

The two reviewers each saw the other's review and are in accord about the two reviews. I would add that I'm particularly sensitive to the caveat/question raised by R1, about the absence of an active control stimulation condition. Its absence is not a deal-breaker, but would require you to soften some of the interpretations as currently phrased. Here are the reviews in their entirety:

R1

Review of eNeuro manucript eN-NWR-0347-17

Prefrontal theta-burst stimulation disrupts the organizing influence of active short-term retrieval on episodic memory

In this study the authors asked whether stimulation of dorsolateral prefrontal cortex could selectively disrupt the influence of short-term active retrieval on hippocampal-dependent long-term memory processes. This is an elegantly-designed study that builds on previous fMRI findings using the same task that helped shape the hypothesis and conclusions. The results are specific and indicate the underlying processes that may be disrupted. I thought this was a great study with a large number of strengths, but that there were a few areas that were lacking in explanation that detract somewhat from the interpretation of the study. I wouldn't necessarily ask them to redo any aspect of the study, but to justify these choices more clearly in the manuscript.

1. The authors don't really give much discussion as to why they chose theta burst stimulation for this study. Given the extensive literature about the role of theta stimulation in memory, including conversion from short-term to long-term memory across studies and species, I wanted a little more clarity about why theta burst was chosen.

2. The experimental stimulation region of interest (DLPFC) was well-justified. Given the authors used an MRI scan to identify the region structurally, I wondered if they had considered using a functional definition (using the same task parameters as in the previous study mentioned, Bridge et al., 2017) and why they had decided not to functionally define the region on a single-subject basis.

3. The control for the DLPFC TBS was sham stimulation. Given that the authors acknowledge the possible effects of DLPFC TBS on the wider network, I also expected a stronger justification for why a control area was not selected. For example, there is a strong claim in the Discussion about the effect shown being selective to the DLPFC, but no comparison regions was stimulated. It is not clear if the effect is specific to the network, or if stimulating, for example, any subregion of prefrontal cortex would have the same effect. The authors do justify the use of sham stimulation from the perspective of peripheral stimulation, but not in terms of the difference in effects on the brain.

4. The active and passive retrieval conditions of the behavioral task seem to differ in not one, but two aspects. First, the participants had to choose the object actively in the ‘active’ condition, whereas it was assigned (surrounded by a red box) in the ‘passive’ condition. Secondly, they had to retrieve the location of the ‘active’ object and not the ‘passive’ object. Why was the object not assigned in both versions of the task, so that the amount of manipulation of object information was the same across both conditions, since spatial memory was the condition of interest?

R2

A well-written and clear manuscript - I have only relatively minor revisions to suggest.

Methods:

1. It would be helpful to include the trial timing information in Figure 1.

2. ‘DLPFC’ is a very broad term - if possible, also state with greater anatomical specificity which region you targeted (i.e. ‘posterior aspect of the superior frontal gyrus’)

3. Last paragraph: It is unclear which objects from each trio were chosen as the reminder cue at test. Were these always the subject-chosen object from the active retrieval trials and the experiment-chosen object from the passive retrieval trials?

Results:

1. There seems to be considerable variability in the effect of DLPFC TMS on immediate spatial recall relative to the baseline session. While this could be due to a mix of factors (variability in the precise TMS target stimulated across participants, etc.), you don't see the same level of heterogeneity in the TMS effects for long-term associative recognition and spatial recall.

a. There may be too few participants to be able to test this, but were those participants whose short-term retrieval performance was lowest after DLPFC TMS also those who showed the largest long-term memory impairments?

2. It is striking that DLPFC TMS seems to impair associative long-term memory more for the actively retrieved items than the passively-restudied items. Based upon the introduction and stated hypotheses, one might expect that the worst associative memory performance would be in the DLPFC stimulation condition after passive restudy. If this comparison is borne out statistically, this point may be worthy of a mention in the discussion. Do you think that strategic active retrieval and restudy processes are employed at the cost of those engaged by default during passive restudy?

3. Figure 2: The figure caption should specify what the error bars reflect (standard error of the mean?). Both positive and negative error bars would allow for simpler visual comparison. Finally, given that the results of the two-way ANOVA are important for the overall interpretation of the results, those could be depicted on the graphs as well.

Discussion:

1. The authors state that “We hypothesize that the DLPFC contribution to long-term memory organization occurred immediately following active retrieval, during the ensuing restudy period” and “During the restudy interval, we have shown that active retrieval influences the pattern of exploratory eye movements subjects engage in, and that these strategic eye movement patterns contribute to long-term memory”.

It seems that one of the main differences between the active retrieval and passive restudy conditions is the feedback/error monitoring component, given that an incorrect object placement in the active retrieval condition would result in the object then being moved to its correct location.

Is there evidence that the DLPFC stimulation does not also affect control processes involved in the active retrieval process itself, such as the choice of which object to report in the manipulation phase? For instance, are the active retrieval manipulation response times the same after DLPFC and sham TMS?

2. A small typo in this sentence: “Indeed, these results are consistent with our fMRI results showing enhanced DLPFC activity following active retrieval, but not during *retrieval*”

## Author Response

Reviewer Response

We thank the reviewers and the editor for their helpful comments. We have addressed the critiques below and edited the manuscript as necessary.

Synthesis Statement for Author (Required):

The two reviewers each saw the other's review and are in accord about the two reviews. I would add that I'm particularly sensitive to the caveat/question raised by R1, about the absence of an active control stimulation condition. Its absence is not a deal-breaker, but would require you to soften some of the interpretations as currently phrased. Here are the reviews in their entirety:

R1

1. The authors don't really give much discussion as to why they chose theta burst stimulation for this study. Given the extensive literature about the role of theta stimulation in memory, including conversion from short-term to long-term memory across studies and species, I wanted a little more clarity about why theta burst was chosen.

Theta-burst stimulation was used because it has been shown to produce relatively long-lasting effects on stimulated regions, including prefrontal cortex. There are only a handful of studies that have investigated the role of theta-patterned stimulation in memory, and we believe that we cite the majority of these studies, in paragraph 3 of the introduction. We are aware of the evidence linking theta-patterned stimulation to memory, particularly in rodent studies of the hippocampus, but the relationship between the extensive literature on theta and hippocampal-dependent memory and noninvasive stimulation is questionable, given the uncertainty about whether theta-burst TMS induces theta-frequency activity in the hippocampal network.

2. The experimental stimulation region of interest (DLPFC) was well-justified. Given the authors used an MRI scan to identify the region structurally, I wondered if they had considered using a functional definition (using the same task parameters as in the previous study mentioned, Bridge et al., 2017) and why they had decided not to functionally define the region on a single-subject basis.

To clarify, we did use a functional definition based on group-level results from Bridge et al., 2017, not a purely anatomical definition. We agree that an ideal experimental design would have included individualized task-based definition of DLPFC for the stimulation target. However, our approach yielded significant results with high behavioral specificity, and so the possible advantages of individualized targeting would seem to be outweighed by the considerable additional resources that would be required.

3. The control for the DLPFC TBS was sham stimulation. Given that the authors acknowledge the possible effects of DLPFC TBS on the wider network, I also expected a stronger justification for why a control area was not selected. For example, there is a strong claim in the Discussion about the effect shown being selective to the DLPFC, but no comparison regions was stimulated. It is not clear if the effect is specific to the network, or if stimulating, for example, any subregion of prefrontal cortex would have the same effect. The authors do justify the use of sham stimulation from the perspective of peripheral stimulation, but not in terms of the difference in effects on the brain.

This is valid point and a weakness of the current study. We cannot say that this particular DLPFC region is selectively involved in this task, while other brain regions are not. Future studies should stimulate different areas of PFC to identify their unique contributions to memory formation. For the purpose of the current study, we have toned down our language in the discussion and added a caveat that we do not show DLPFC selectivity in this task. However, based on this study, we can say that the effects of DLPFC on task performance are highly specific to long-term memory in the active retrieval condition.

4. The active and passive retrieval conditions of the behavioral task seem to differ in not one, but two aspects. First, the participants had to choose the object actively in the ‘active’ condition, whereas it was assigned (surrounded by a red box) in the ‘passive’ condition. Secondly, they had to retrieve the location of the ‘active’ object and not the ‘passive’ object. Why was the object not assigned in both versions of the task, so that the amount of manipulation of object information was the same across both conditions, since spatial memory was the condition of interest?

It is true that the active manipulation varies in both the selection and retrieval process. We modeled our study after the Bridge et al. 2017 study, which included both selection and retrieval in the active condition. The main goal of the manipulation was to cause one object to be actively manipulated, and we defined that manipulation as selection and retrieval. We chose both selection and retrieval to strengthen the active engagement with one object in the active condition.

R2

Methods:

1. It would be helpful to include the trial timing information in Figure 1.

We have added the trial timing to the Figure 1 caption.

2. ‘DLPFC’ is a very broad term - if possible, also state with greater anatomical specificity which region you targeted (i.e. ‘posterior aspect of the superior frontal gyrus’)

Thank you for the suggestion. We have provided a more specific definition of the targeted area in the MRI section of the Methods.

3. Last paragraph: It is unclear which objects from each trio were chosen as the reminder cue at test. Were these always the subject-chosen object from the active retrieval trials and the experiment-chosen object from the passive retrieval trials?

The reminder cue at test could be any object from the trio. Half the time it was the manipulated object (actively retrieved object/passively retrieved object) and half the time it was one of the non-manipulated objects from the trio. The tested object was always a non-manipulated object. We clarified this point in the paragraph.

Results:

1. There seems to be considerable variability in the effect of DLPFC TMS on immediate spatial recall relative to the baseline session. While this could be due to a mix of factors (variability in the precise TMS target stimulated across participants, etc.), you don't see the same level of heterogeneity in the TMS effects for long-term associative recognition and spatial recall.

a. There may be too few participants to be able to test this, but were those participants whose short-term retrieval performance was lowest after DLPFC TMS also those who showed the largest long-term memory impairments?

Due to your request below (Point 3), we re-examined the error bars for the graph and noticed an error in the short-term retrieval graph (we selected the wrong standard error for this figure). As you can now see in Figure 2, the variability in short-term retrieval is evident for both groups, but just to highlight the y-axis, the variability is relatively small (SE=~0.03).

As you requested, we did compare long-term memory performance in the Stim group based on a median split of the short-term retrieval change scores, which left only 7 participants in each group for this analysis (Stim group: high vs. low short-term retrieval accuracy change). Change in short-term memory accuracy across sessions did not vary with change in long-term associative recognition memory [t(13)=0.33, p=0.75]. Mean long-term associative recognition change for subjects with lowest short-term memory accuracy was -0.01 (SE=0.04), whereas mean associative recognition change for subject with highest short-term memory accuracy in the Stim group was -0.03 (SE=0.04).

2. It is striking that DLPFC TMS seems to impair associative long-term memory more for the actively retrieved items than the passively-restudied items. Based upon the introduction and stated hypotheses, one might expect that the worst associative memory performance would be in the DLPFC stimulation condition after passive restudy. If this comparison is borne out statistically, this point may be worthy of a mention in the discussion. Do you think that strategic active retrieval and restudy processes are employed at the cost of those engaged by default during passive restudy?

We hypothesized that DLPFC stimulation would selectively alter long-term memory in the active condition, due to the previous fMRI finding (Bridge et al., 2017) that implicated DLPFC in organizing event information in the active, but not the passive condition. Indeed, subjects are still able to remember the object-location associations in the passive condition, so some organization of the event information must occur in that condition. However, we hypothesize that memory in the passive condition could arise from various non-specific factors, including subject-specific strategies, semantic associations, salience of the objects, attentional variation, etc. Here, we used an active retrieval manipulation to demonstrate a specific process in which DLPFC is involved in organizing event information (i.e. active retrieval), and we show that stimulation of DLPFC impairs this specific organizational process during learning.

3. Figure 2: The figure caption should specify what the error bars reflect (standard error of the mean?). Both positive and negative error bars would allow for simpler visual comparison. Finally, given that the results of the two-way ANOVA are important for the overall interpretation of the results, those could be depicted on the graphs as well.

We have added positive and negative error bars to the figures. We have also specified that the error bars reflect standard error of the mean in the figure caption. The data presented in the figures do correspond to the two-way ANOVA that was performed, and so we are unsure what additional information the reviewer would like to see added to the figures.

Discussion:

1. The authors state that “We hypothesize that the DLPFC contribution to long-term memory organization occurred immediately following active retrieval, during the ensuing restudy period” and “During the restudy interval, we have shown that active retrieval influences the pattern of exploratory eye movements subjects engage in, and that these strategic eye movement patterns contribute to long-term memory”.

It seems that one of the main differences between the active retrieval and passive restudy conditions is the feedback/error monitoring component, given that an incorrect object placement in the active retrieval condition would result in the object then being moved to its correct location.

Is there evidence that the DLPFC stimulation does not also affect control processes involved in the active retrieval process itself, such as the choice of which object to report in the manipulation phase? For instance, are the active retrieval manipulation response times the same after DLPFC and sham TMS?

We examined the change in object-selection times across sessions for the Active and Passive condition separately. *Values are negative because reaction times decreased across sessions, irrespective of stimulation condition.

In the Active condition, change in object-selection times did not differ across Stim and Sham groups [Stim: M=-286.20 ms, SE=78.42; Sham: -192.55, SE=49.50; t(13)=1.01, p=0.32]. Change in object-selection times also did not differ across groups in the Passive condition [Stim: -249.98, SE=52.87; Sham: -263.50, SE=68.83; t(13)=0.16, p=0.88]. Given no significant effects on object-selection reaction times, these results indicate that DLPFC stimulation did not affect control processes involved in selecting an object during the manipulation phase.

2. A small typo in this sentence: “Indeed, these results are consistent with our fMRI results showing enhanced DLPFC activity following active retrieval, but not during *retrieval*”

Thank you for pointing out this confusing sentence. Although this is not a typo, we have highlighted ‘following’ active retrieval and ‘during’ retrieval to emphasize that we hypothesize that effects of DLPFC stimulation effects on task performance occurred due to processing affected after the active retrieval manipulation, but it did not necessarily alter performance of the short-term active retrieval manipulation itself (i.e. during retrieval). To clarify, in the task, subjects Initially Study the Objects, then Actively retrieve one object, and finally Restudy the objects. In the referenced fMRI study, the authors found that DLPFC activity increased during the Restudy period following Active retrieval compared to the Restudy period following Passive restudy. On the other hand, fMRI activity in DLPFC did not differ across Active Retrieval and Passive Restudy conditions during the manipulation period.
